# Thermal diffusivity measurement of spherical gold nanofluids of different sizes/concentrations

**DOI:** 10.1186/1556-276X-7-423

**Published:** 2012-07-30

**Authors:** Gerardo A López-Muñoz, José A Pescador-Rojas, Jaime Ortega-Lopez, Jaime Santoyo Salazar, J Abraham Balderas-López

**Affiliations:** 1 Química Aromática SA, Río Grande S/N, Col. Santa Catarina Acolman, México State, CP, 55875, Mexico; 2 UPIBI-IPN, Av. Acueducto S/N, Col. Barrio la Laguna Ticomán, México City, CP, 07340, Mexico; 3 Physics Department, CINVESTAV, Av. IPN 2508, Col. San Pedro Zacatenco, México City, CP, 07360, Mexico; 4 Biotechnology Department, CINVESTAV, Av. IPN 2508, Col. San Pedro Zacatenco, México City, CP, 07360, Mexico

**Keywords:** Gold nanoparticles, Nanofluids, Photoacoustic, Thermal diffusivity

## Abstract

In recent times, nanofluids have been studied by their thermal properties due to their variety of applications that range from photothermal therapy and radiofrequency hyperthermia (which have proven their potential use as coadjutants in these medical treatments for cancer diseases) to next-generation thermo-fluids. In this work, photoacoustic spectroscopy for a specific study of thermal diffusivity, as a function of particle size and concentration, on colloidal water-based gold nanofluids is reported. Gold nanoparticles were synthetized in the presence of hydroquinone through a seed-mediated growth with homogenous sizes and shapes in a range of 16 to 125 nm. The optical response, size and morphology of these nanoparticles were characterized using ultraviolet–visible spectroscopy and transmission electron microscopy, respectively. Thermal characterizations show a decrease in the thermal diffusivity ratio as the nanoparticle size is increased and an enhancement in thermal diffusivity ratio as nanoparticle concentration is added into the nanofluids. Compared with other techniques in the literature such as thermal lens and hot wire method, this photoacoustic technique shows an advantage in terms of precision, and with a small amount of sample required (500 μl), this technique might be suitable for the thermal diffusivity measurement of nanofluids. It is also a promising alternative to classical techniques.

## Background

Thermal properties of nanofluids, which are mixtures of nanomaterials suspended in an organic or inorganic base fluid, are especially interesting due to their variety of applications that range from photothermal therapy and radiofrequency hyperthermia (which have proven their potential use as coadjutants in these medical treatments for cancer diseases [1,2]) to next-generation thermo-fluids. Recently, it has been found that nanofluids exhibit higher thermal conductivity and thermal diffusivity than base fluids themselves. Thermal research in nanofluids has been mainly focused in thermal conductivity measurements [3-5], but in recent years, other techniques have been developed for thermal diffusivity measurements in nanofluids, such as the hot-wire technique and the thermal lens spectrometry [6-8]. However, these methodologies are limited due to the difficulty in achieving accurate results, the complexities in their theoretical model and the amount of sample required for measurements.

In this article, an aqueous synthesis of gold nanoparticles in the presence of hydroquinone through a seed-mediated growth is presented. Also, the study of thermal diffusivity in gold nanofluids as a function of particle size (16–125 nm) and concentration by photoacoustic spectroscopy opens new horizons for the thermal research area in nanofluids.

## Methods

### Hydroquinone synthesis of gold nanoparticles

Typical synthesis of gold nanoparticles by the chemical reduction of gold chloride using sodium citrate can only produce quality particles up to 50 nm in size; beyond which, they are poly-dispersed and non-spherical [9]. The reduction of gold chloride onto nanoparticle seeds (gold nanoparticles, <20 nm) together with hydroquinone as a selective reducing agent improves these obtained particles with homogenous sizes and shapes in a size range of 50 to 200 nm [10,11].

Gold nanoparticle seeds were synthetized by sodium citrate reduction. A solution of gold chloride is brought to boil and immediately followed by the addition of a solution of sodium citrate. The solution is removed from heat once nanoparticle maturation is completed, as indicated by the colour transition.

Large-diameter gold nanoparticles were synthetized by hydroquinone method, using consistent concentrations of gold chloride, sodium citrate and hydroquinone but decreasing the number of seeds, which results in the growth of larger nanoparticles. The addition of less seeds results in larger gold nanoparticle diameters. The different size nanofluids were centrifuged at 6,000 rpm for 30 min and re-dispersed with high-performance liquid chromatography (HPLC) water for a final concentration of 0.1 mg/ml.

For concentrated nanofluids, stock solutions of gold nanoparticles synthetized by hydroquinone method were centrifuged at 6,000 rpm for 30 min for final concentrations of 1 mg/ml. The concentrated solutions were re-dispersed with HPLC water to obtain different gold nanoparticle concentrations.

All chemicals used were of analytical grade from Sigma-Aldrich Corporation (St. Louis, MO, USA) and were used as received; HPLC water was used in all the gold nanoparticles’ synthesis.

### Basic photoacoustic theoretical scheme

As shown elsewhere [12], the photoacoustic signal in transmission configuration, assuming sample thickness *L* as the only variable (with fixed modulation frequency *f*) in the liquid’s sample thermally thick regime, can be expressed as follows:

(1) $$\delta P=E\exp\left(-\sigma_{\text{s}}L\right)\text{,}$$

where *E* is a complex constant and *σ*_s_ = (1 + *i*)(π*f/α*_s_)^1/2^, with *α*_s_ as the sample’s thermal diffusivity. The amplitude ∣*δP*∣ and phase Ф, of this equation can be written as follows:

(2) $$\left|\delta P\right|=\left|E\right|\exp\left(-\sqrt{\frac{\text{π}f}{\alpha_{\text{s}}}}L\right)\text{,}$$

(3) $$\Phi =\Phi_{0}-\sqrt{\frac{\pi f}{\alpha_{\text{s}}}}L\text{.}$$

The signal phase Ф is a linear function of the sample thickness with the slope given by the following:

(4) $$B={\left(\frac{\pi f}{a_{\text{s}}}\right)}^{\frac{1}{2}}\text{,}$$

from which the sample’s thermal diffusivity can be obtained.

### Experimental setup

**Figure 1:**
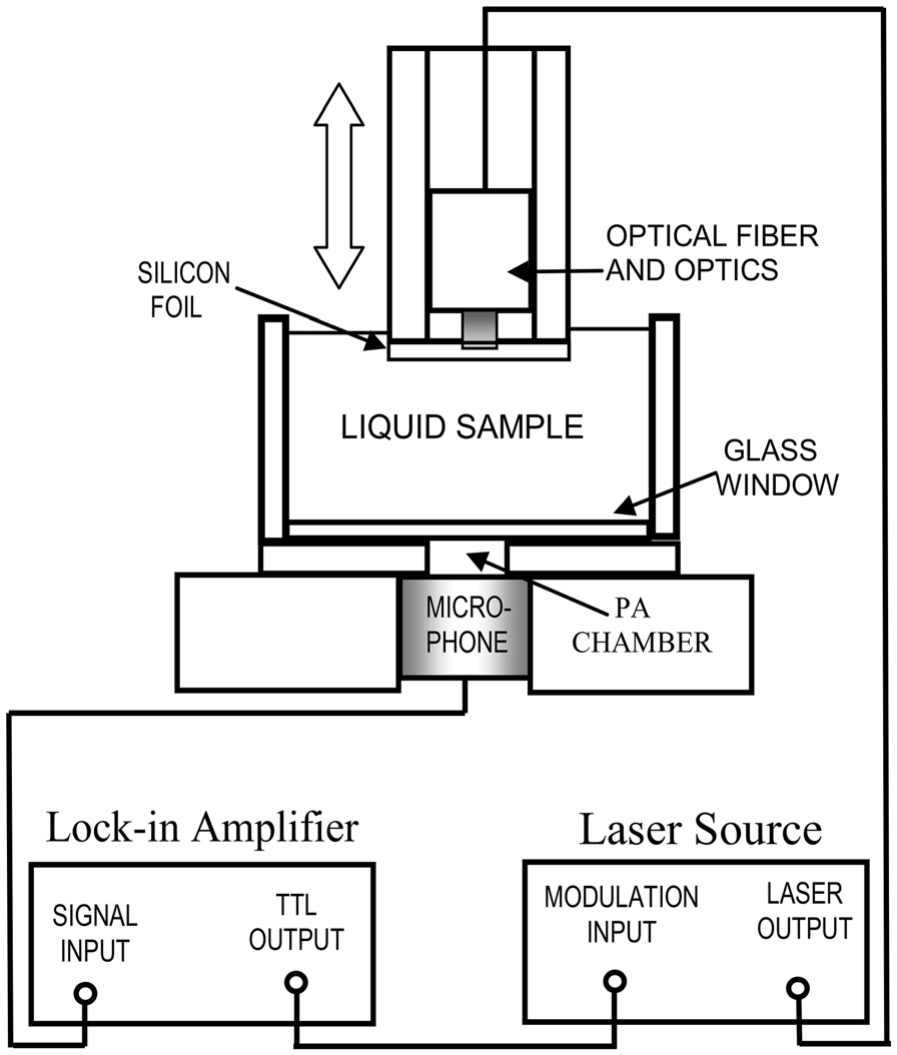
Cross section of the photoacoustic experimental system.

A transversal section of the photoacoustic experimental setup for thermal diffusivity measurements is shown in Figure 1. The photoacoustic cell consisted of a cylindrical cavity made in a stainless steel body and communicated with a commercial electret microphone (Panasonic, Kadoma, Osaka, Japan), model WM-61A. The resultant signals detected by the microphone were processed by a lock-in amplifier model SR830 (Stanford Research, Menlo Park, CA, USA) for amplification and de-modulation; the transistor-transistor logic output of the lock-in was used for the modulation control of a 660-nm laser diode system (Qioptiq Photonics Ltd. (formerly Point Source Ltd.), Hamble, Hampshire, UK) model IFLEX-2000 at a fix modulation frequency of 2 Hz.

The photoacoustic signal was recorded as a function of the sample thickness, taking 16 experimental points from a relative sample thickness *l*_0_ at 10-μm steps using a micro-linear actuator model T-NA08A50 (Zaber Technologies, Inc., Vancouver, Canada). Linear fits were done for the photoacoustic phase to obtain parameter *B*, as described in the theoretical section, from which the sample’s thermal diffusivity was obtained by means of the relation *α*_*s*_ = 2π/*B*^2^. Measurements were performed at room temperature 25 ± 2°C.

## Results and discussion

### Nanoparticle characterization

**Figure 2:**
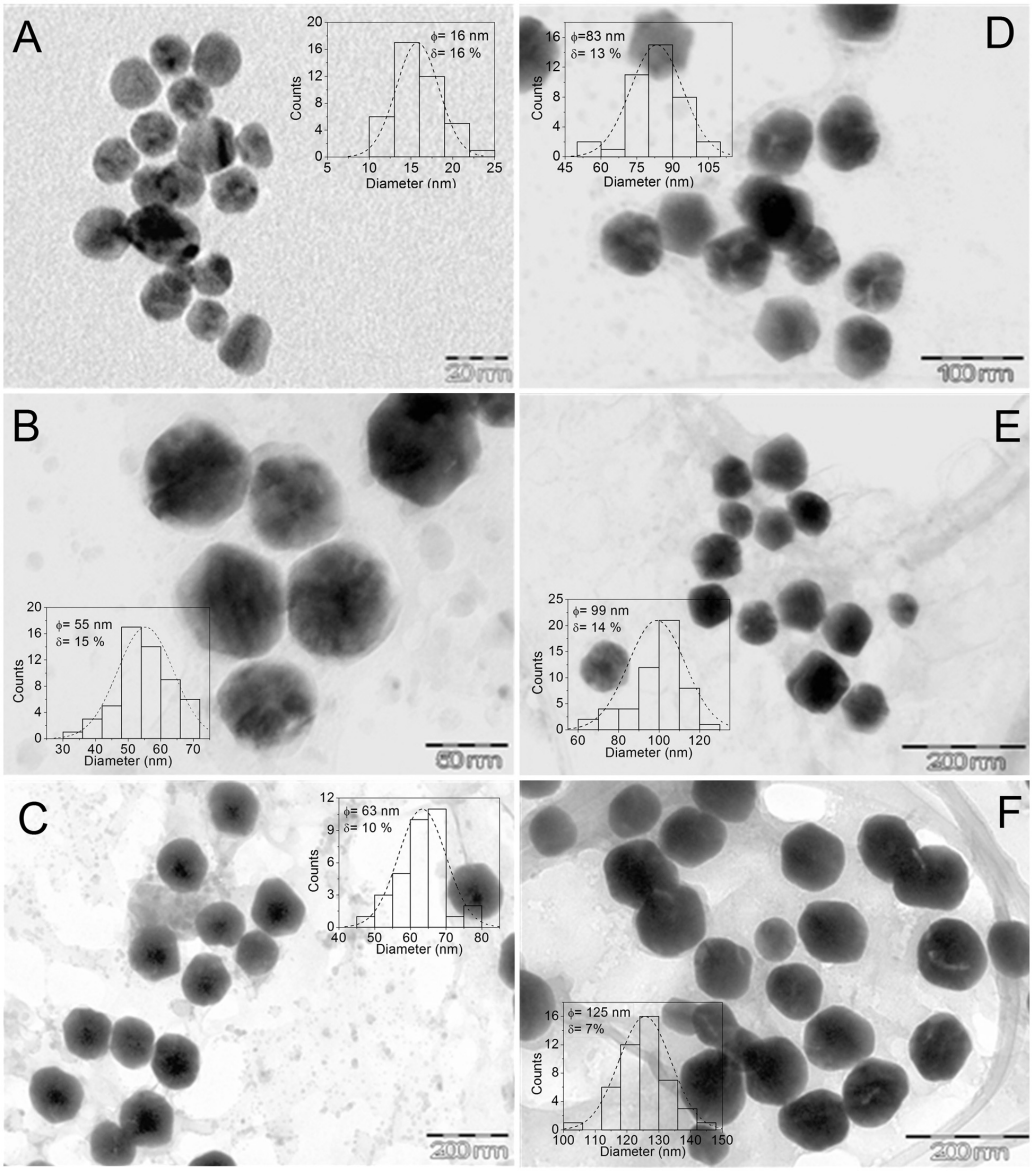
** Representative TEM images and size distributions of the six particle sizes obtained.** (**A**) 16 (seeds), (**B**) 55, (**C**) 63, (**D**) 83, (**E**) 99, and (**F**) 125 nm.

The particle size, shape/morphology and distribution of nanoparticles in the base medium were determined by transmission electron microscopy (TEM) with a JEOL JEM2010 equipment (JEOL Ltd., Akishima, Tokyo, Japan) at 200 kV and 106 μA. Samples were prepared by adding 50 μl of the stock nanoparticle solution onto 200-mesh carbon-coated copper grids to obtain TEM images. In Figure 2, TEM images show a larger diameter of the gold nanoparticles by decreasing the number of seeds, as it was expected. The dispersion of gold nanoparticles has a median size distribution near 12% for all the samples, including large-diameter gold nanoparticles. This is an advantage if compared with the citrate method, in which it is only possible to produce monodispersed and spherical gold nanoparticles of up to 50 nm in diameter.

**Figure 3:**
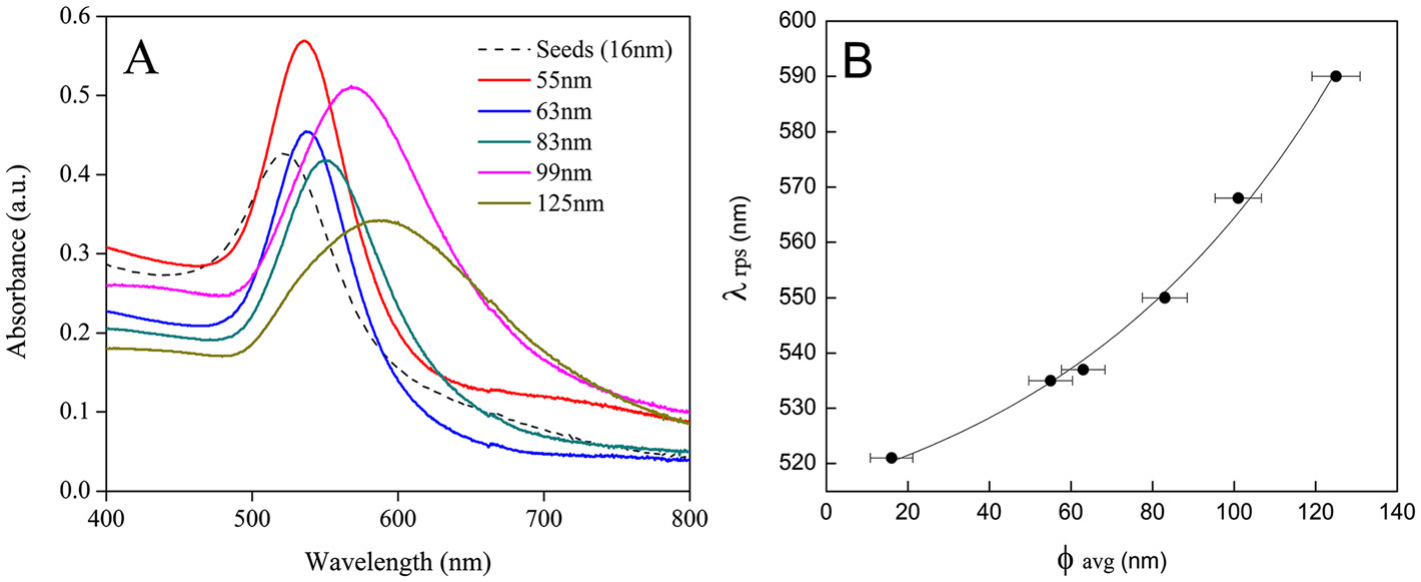
** UV–vis absorption spectra and absorption shift of gold nanoparticles.** (**A**) UV–vis absorption spectrum of the synthetized gold nanoparticles. (**B**) Shift in absorption λ_rps_ of gold nanoparticles with particle size.

The absorption spectra of synthetized nanoparticles were measured as 1:2 dilutions (stock to H_2_O) using an ultraviolet–visible (UV–vis) spectrophotometer (8453 Agilent Technologies, Inc., Santa Clara, CA, USA). Figure 3A shows the UV–vis absorption spectra of the different size gold nanofluids studied. As expected, the increase in gold nanoparticle diameters resulted in a longer absorption λ_*max*_ values (Figure 3B), according to Mie’s theory [13,14].

#### *Thermal diffusivity measurement*

Thermal diffusivity values of 9.31 × 10^−4^ ± 0.04 × 10^−4^ cm^2^·s^−1^ for glycerol (J.T. Baker Reagent Chemicals, Phillipsburg, NJ, USA) and values of 14.35 × 10^−4^ ± 0.04 × 10^−4^ cm^2^·s^−1^ for HPLC grade water (Sigma-Aldrich Corporation) were obtained by the photoacoustic system as reference samples for system calibration with a coefficient of variation near 2% to the reported values in the literature [15,16]. The resulting thermal diffusivity values for the different sizes and concentrations of gold nanofluids are summarized in Tables 1 and 2, respectively; these are average values over five measurements of each sample taking the standard deviation in measurements as the uncertainty.

**Table 1:** Thermal diffusivity of gold nanofluids for different nanoparticle diameters for constant nanoparticle concentration of 0.1 mg/ml

**Particle size (nm)**	***α*(10**^**−4**^ **cm**^**2**^**·s**^**−1**^**) ± 0.04**
16	14.98
55	14.71
63	14.63
83	14.53
99	14.51
125	14.46

**Table 2:** Thermal diffusivity of gold nanofluids at different nanoparticle concentrations

**Concentration**	***α***_**55 nm**_**(10**^**−4**^ **cm**^**2**^**·s**^**−1**^**) ± 0.04**	***α***_**83nm**_**(10**^**−4**^ **cm**^**2**^**·s**^**−1**^**) ± 0.04**
0.2 mg/ml	14.74	14.56
0.4 mg/ml	14.81	14.59
0.6 mg/ml	14.85	14.64
0.8 mg/ml	14.91	14.69
1 mg/ml	14.99	14.76

#### *Nanoparticle size*

**Figure 4:**
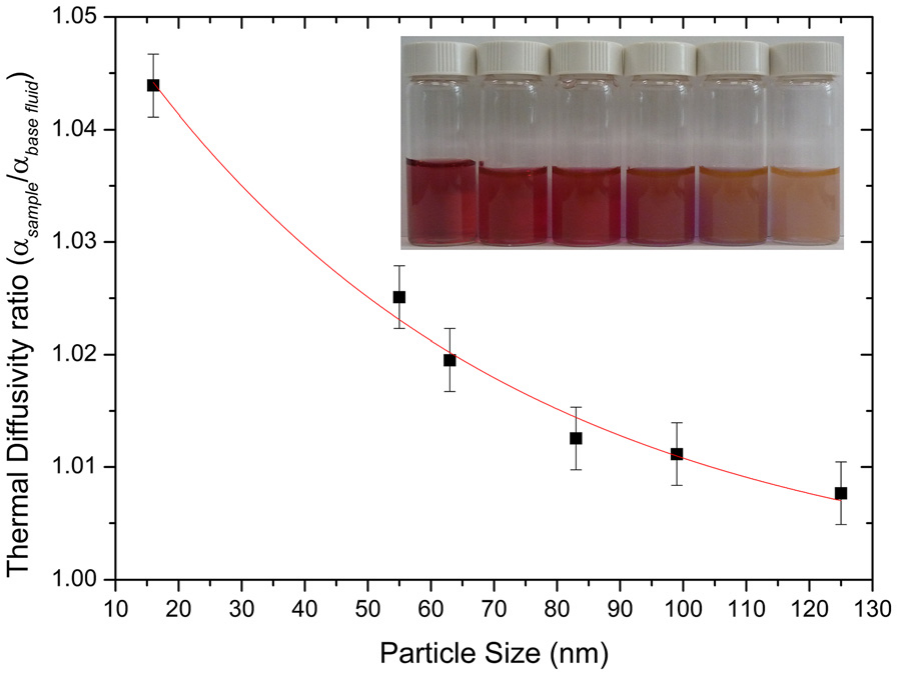
** Thermal diffusivity decrease ratio with gold nanoparticle size.** The nanoparticle concentration was 0.1 mg/ml. The inset shows (left to right) the different size nanofluids of 16 (seeds), 55, 63, 83, 99 and 125 nm.

Figure 4 shows the thermal diffusivity values of gold colloid nanofluids as a function of nanoparticle size; it shows that if the nanoparticle concentration is constant (0.1 mg/ml), then the thermal diffusivity ratio (*α*_sample_/*α*_base fluid_) of nanofluids increases as the nanoparticle diameter is reduced. This behaviour can be explained as follows: as the particle size decreases, the effective surface area increases, and the Brownian motion of nanoparticles enhances convective heat transfer mechanisms; thermal diffusivity and thermal conductivity of nanofluids increase as a result. However, for large-diameter nanoparticles, the surface area and the Brownian motion both decrease, and as a consequence, there is no enhancement of thermal diffusivity and thermal conductivity by convection [3].

#### *Concentration*

**Figure 5:**
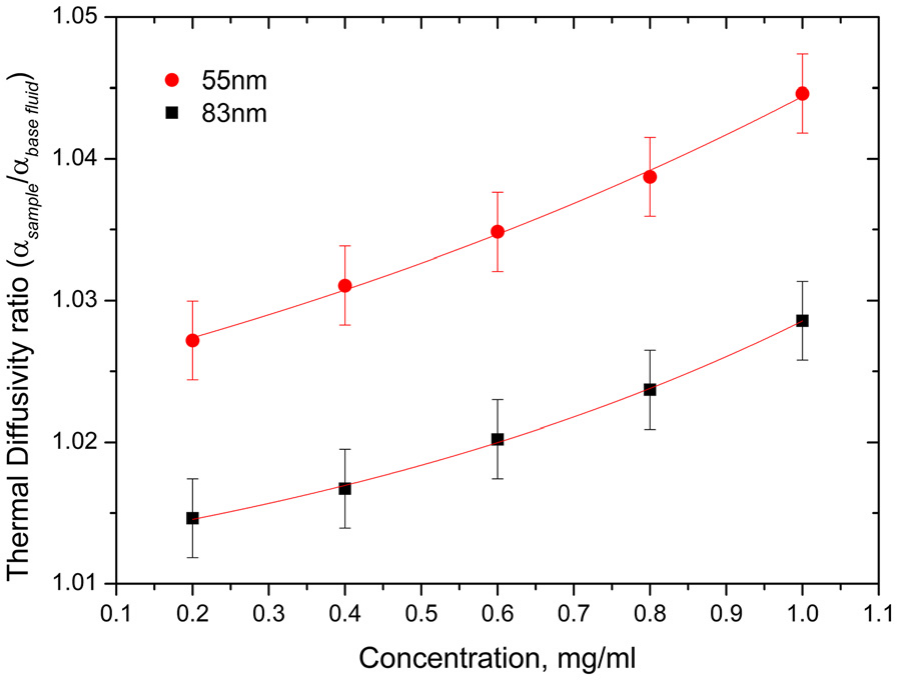
** Thermal diffusivity enhancement ratio with gold nanofluid concentration.** The nanoparticle average diameters were 55 and 83 nm.

Figure 5 shows the nanoparticle concentration dependency of the nanofluid thermal diffusivity for 55 and 83 nm size nanoparticles; as the nanoparticle concentration increases, the thermal diffusivity ratio (*α*_sample_/*α*_base fluid_) of nanofluids increases; the results depict similar behaviours reported in the literature for metal nanofluids by thermal lens spectrometry, and photopyroelectric and the hot wire methods [7,8,17,18]. This dependency can be explained as follows: the dissociation of the surfactant (sodium citrate) may intensify the enhancement of thermal properties by inducing electrostatic repulsion among the suspended nanoparticles resulting in stabilized suspensions. Moreover, with the volumetric increase of nanoparticles, the specific heat of nanofluids decreases; as a consequence, the thermal diffusivity of the colloidal suspension increases [18]. The enhancement of convection and the decrease in specific heat increase the thermal diffusivity ratio of 55 nm concentrated nanofluids compared with 83 nm concentrated nanofluids.

## Conclusions

The present study investigates the effect of concentration and size of gold nanoparticles on the thermal diffusivity of gold nanofluids prepared by hydroquinone method through a seed-mediated growth by photoacoustic spectroscopy. The thermal diffusivity ratio changed inversely with the nanoparticle size; this effect was studied in a size range of 16 to 125 nm for gold nanofluids. The thermal diffusivity ratio has been found to increase with concentration; this effect was studied in a range of 1 to 0.2 mg/ml for gold nanofluids. The experimental data for thermal diffusivity ratio as a function of concentration and size for gold nanofluids depict similar behaviours in enhancement ratio compared to thermal conductivity reports and other techniques in the literature such as thermal lens and hot wire method with high accuracy, and with a small amount of sample required (500 μl), photoacoustic spectroscopy might be suitable for thermal diffusivity measurement of nanofluids and being a promising alternative to classical techniques.

## Abbreviations

HPLC: high-performance liquid chromatography; TEM: transmission electron microscopy; UV–vis: ultraviolet–visible.

## Competing interests

The authors declare that they have no competing interests.

## Authors’ contributions

GALM and JAPR prepared all the samples, set up the photoacoustic method and measured and analysed the UV–vis and photoacoustic data. JSS measured and analysed the TEM data. JOL and JABL designed the experiments and wrote the manuscript. All authors read and approved the final manuscript.
